# Science in the News

**DOI:** 10.1371/journal.pbio.0040055

**Published:** 2006-02-14

**Authors:** Hemai Parthasarathy

## Abstract

Do press embargoes hurt or help the reporting of science news?

An article in *PLoS Biology* was downloaded more than 40,000 times in the span of a single November week. It was not a cure for cancer, it was not the discovery of a new link in human evolution. It was a paper entitled “Ultrasonic Songs of Male Mice” by Tim Holy and Zhongsheng Guo, and, as the title aptly indicates, it described the songlike vocalizations of laboratory mice. The unprecedented number of downloads was no doubt driven by the widespread press attention accorded this article. We lost count of the number of outlets that covered the story, but they ranged from such venerable broadsheets as *The Guardian* and *The New York Times* to postings on slashdot.org. The paper's reviewers and editors were pleased to see it published in *PLoS Biology* as an important piece of work in the field, but as the lead author himself commented when inundated by requests for interviews from journalists, “[W]hile I'm proud of the work, it's certainly a disproportionate amount of attention given how many other interesting things there are in science.”

News of scientific breakthroughs simultaneously hits the presses around the world in a carefully orchestrated process. Science news, in general, is not like other news; it is seldom reported as it happens. There are, of course, genuine scientific news events—the first lunar landing, the first photographs from the Hubble telescope—moments that prompt applause for technological achievement and give snapshots of the first data that will ultimately lead to a better understanding of our world. But, the news report rarely follows on the heels of the “eureka moment” of scientific discovery.

Instead, science journalists typically learn about scientific breakthroughs from coordinated press releases issued by the scientific journal or by the researcher's academic institution. Press releases are usually made available a week or so before publication, giving journalists time to interview authors and their peers and research the context of the story, knowing that the story will not be scooped by another journalist. Many journals will not even review an article if the press has already covered the story, or will threaten to reject articles if the story is somehow leaked to the press before publication. Authors, therefore, are usually reluctant to talk to the press without assurance that the story will not come out prior to publication of the research article.

Scientific journals benefit from the press-embargo system because it maximizes and coordinates publicity for the journal—essentially providing free marketing with a global reach. Embargoes also ensure that the story is reported on the same day by different media outlets, since no single outlet can get the jump on everyone else. Although we will never penalize a potential author if press attention has preceded publication of the primary research, *PLoS Biology* maintains a press-release system and we ask that journalists respect our embargoes. We support this system because our content is freely available immediately upon publication, and it is our hope that those particularly interested in a science news story will be able to get the facts from the actual article, rather than relying on secondary sources. Indeed, our most gratifying feedback on the singing-mouse paper came not from a scientist, but from a teacher:

I just listened to a mouse song online, a figure in a paper you published, and I wanted to tell you how grateful I am for your journal. I am a middle-school science teacher. I do not have the funds to subscribe to the traditional science journals. Tomorrow my students will hear the same mouse song I listened to and I am sure they will be as enchanted and interested as I am. The idea of open access to original research papers is very exciting to someone in my position … I can assure you that the availability of research papers will benefit the future of scientific research by providing motivation and stimulation for millions of fledgling scientists.

Many argue, however, that the embargo system creates a false understanding of the scientific process and suppresses true investigative reporting of science. The rationale for not reporting science news as the discoveries unfold is, of course, that we rely on the peer-review system for validation. Although peer-review offers no guarantees, it would be irresponsible for a scientist to publicize the potency of a new drug in the fight against cancer on the strength of preliminary experiments. But, even after acceptance, with the stamp of approval given by peers that the science therein is trustworthy, several weeks or even months can pass before the paper is published and the embargo lifted. Thus, the moment that science becomes news is somewhat arbitrary.

Perhaps science news, then, should not be considered a special case of the news. After all, unpublished, un-peer-reviewed science is covered in the news when it is presented at big scientific congresses. And the number of Web sites presenting un-peer-reviewed science abound. Lab blogs may not yet have made a widespread appearance; however, nothing but ingrained traditions of ascribing priority prevent scientists from sharing their data as they acquire it—as they already do, for instance, when sequencing genomes. Perhaps professional journalists covering science should be encouraged to do so when they become aware of a story, carrying the responsibility for conveying to the public the context and stage of the research project. As with everything we do, PLoS is guided by the interests of the scientific community and members of the public who support research. In our ongoing effort to advance these interests, we welcome feedback on our embargo policy.

